# Supplementary material for: Exploring the Mechanisms of Iron Overload-Induced Liver Injury in Rats Based on Transcriptomics and Proteomics
Source: Biology (Basel). 2025 Jan 16;14(1):81. doi: 10.3390/biology14010081 (PMC11761193; doi:10.3390/biology14010081)
Supplement: Supplementary file 1 [file biology-14-00081-s001.zip › biology-3382499-Supplementary materials.pdf]

Table S1

The list of antibodies

Antibodies	Source	Catalogue NO.	Dilution
Primary antibody			
HO-1	Proteintech	10701-1-AP	1:100~1:1000
Tubulin-α	Bioworld	BS1699	1:10000
Secondary antibody			
Goat anti-Rabbit IgG (H+L)-HRP	Bioworld	BS13278	1:10000
Goat anti-Rabbit IgG (H+L)-Alexa Fluor® 488	Abcam	Ab150077	1:1000

Table S2

The list of antibodies

Target genes	Primer sequences (5' to 3')	GenBank No.
Ho-1	F: AGCCTGGTTCAAGATACTACC<br>R: GGGGCCAACACTGCATTTAC	NM_012580.2
Lnc286.2	F: CTGGGGCAAGATTTATCTGGAACTA<br>R: GTTCTGGCCTGCATTCAAATACTTC	NM_001191756.2
Lnc362.2	F: GCCCAGAACTACGACATAAACGATA<br>R: GCTTCTTCTTGGCTTCCAGGTC	NM_001195503.2
Gapdh	F: GGAGAAACCTGCCAAGTATGA<br>R: AATGGGAGTTGCTGTTGAAGT	NM_017008.4

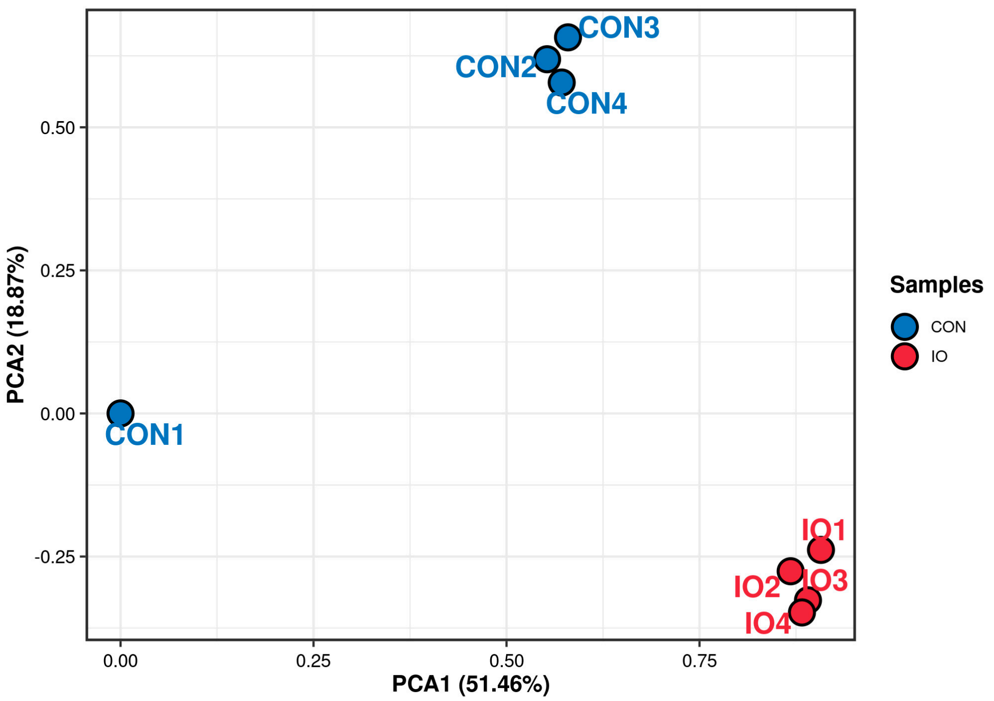

Figure S1. Principal components analysis (PCA) of proteins. CON: control, IO: iron overload, n=4.

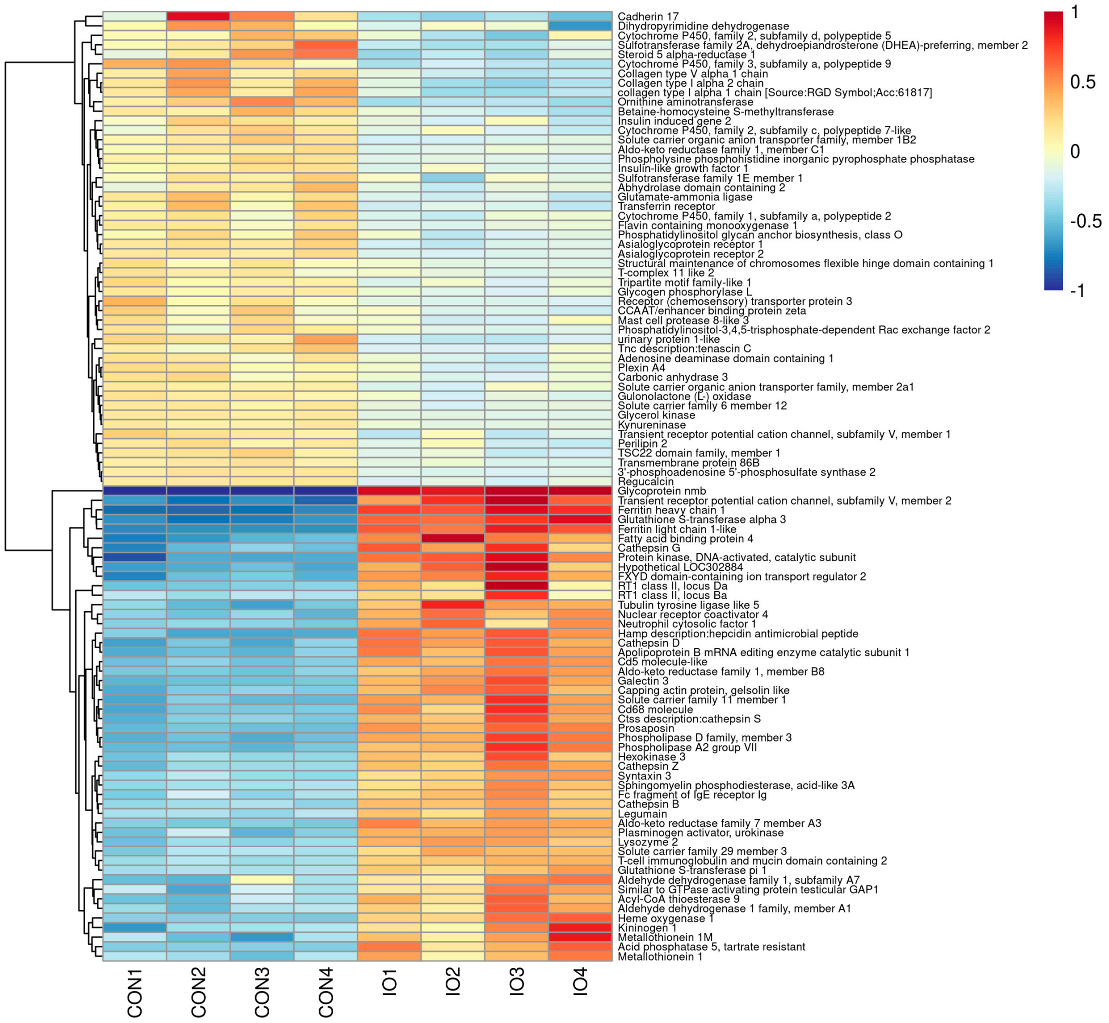

Figure S2. Hierarchical clustering heatmap of the top 50 up-regulated and top 50 down-regulated proteins. CON: control, IO: iron overload, n=4.

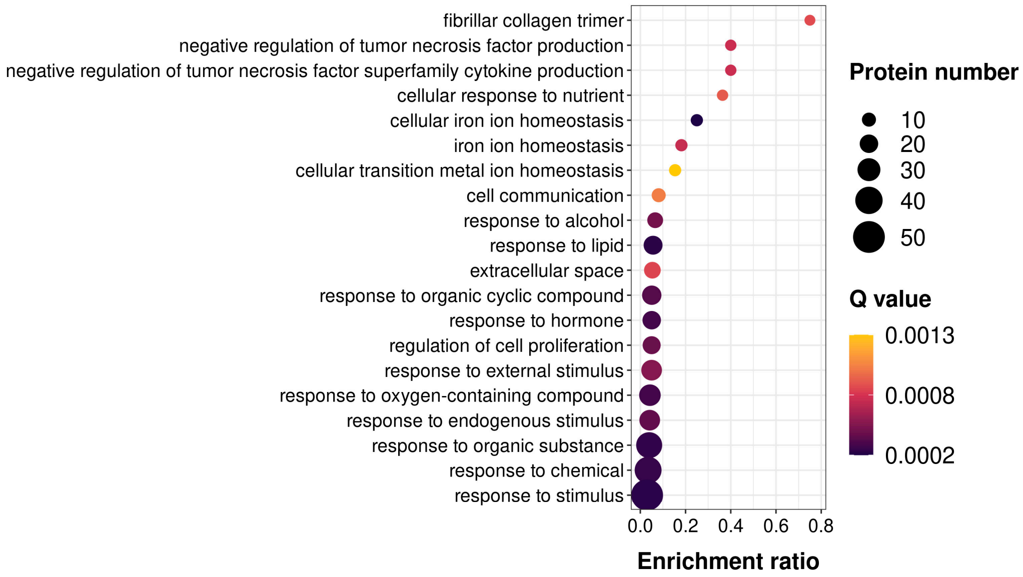

Figure S3. Top20 in GO enrichment analysis of 50 up-regulated and 50 down-regulated proteins.

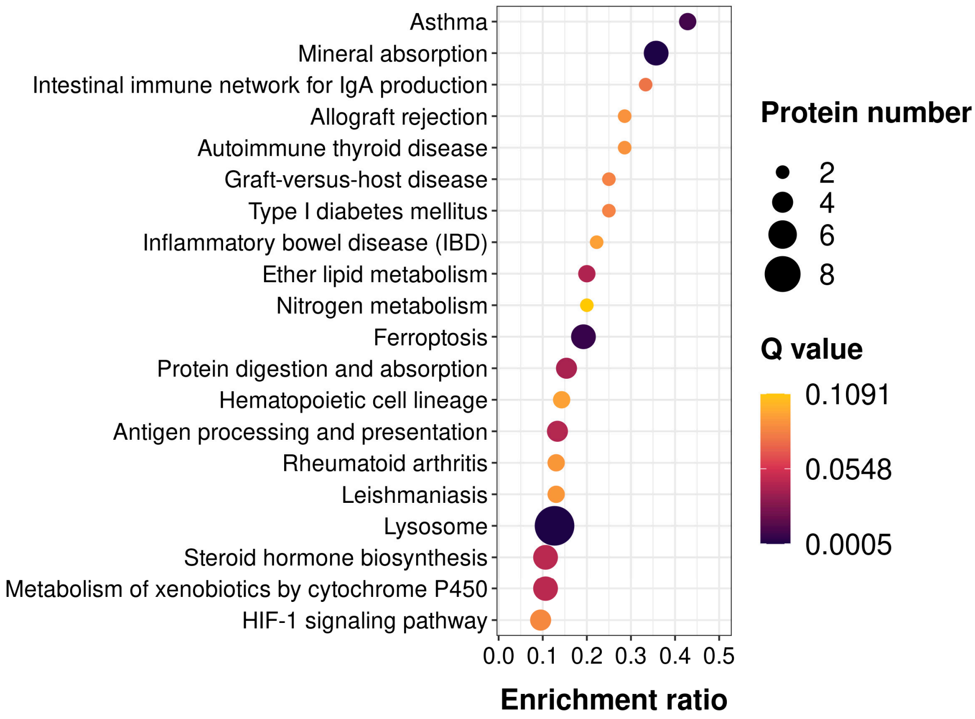

Figure S4. Top20 in KEGG enrichment analysis of 50 up-regulated and 50 down-regulated proteins.

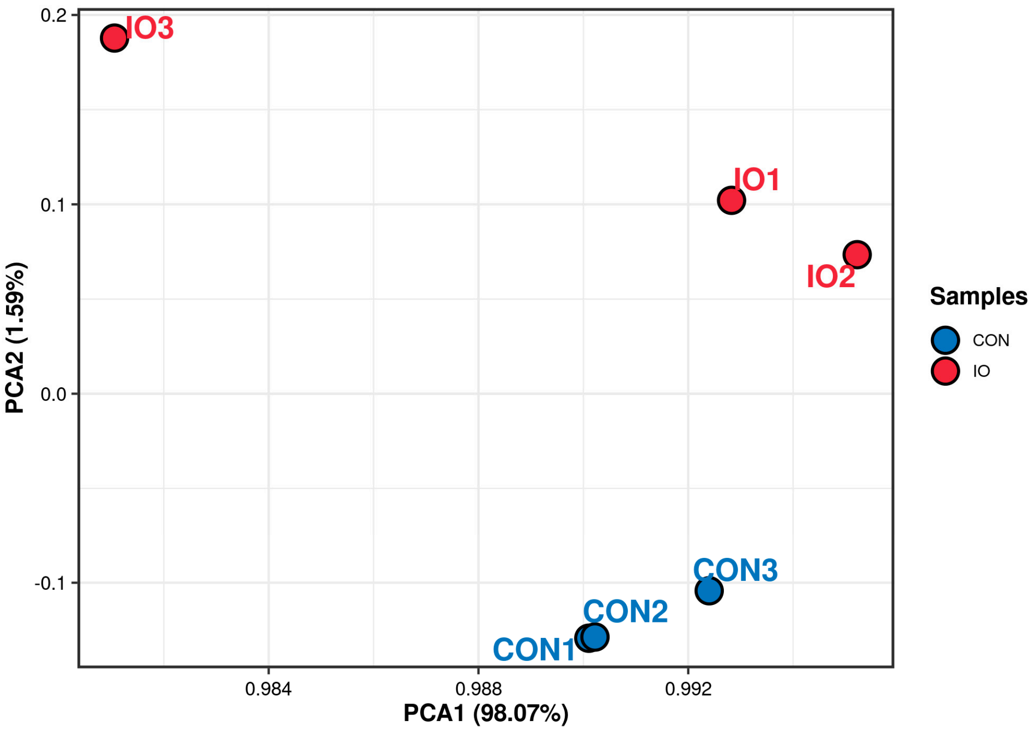

Figure S5. Principal components analysis (PCA) of mRNAs. CON: control, IO: iron overload, n=3.

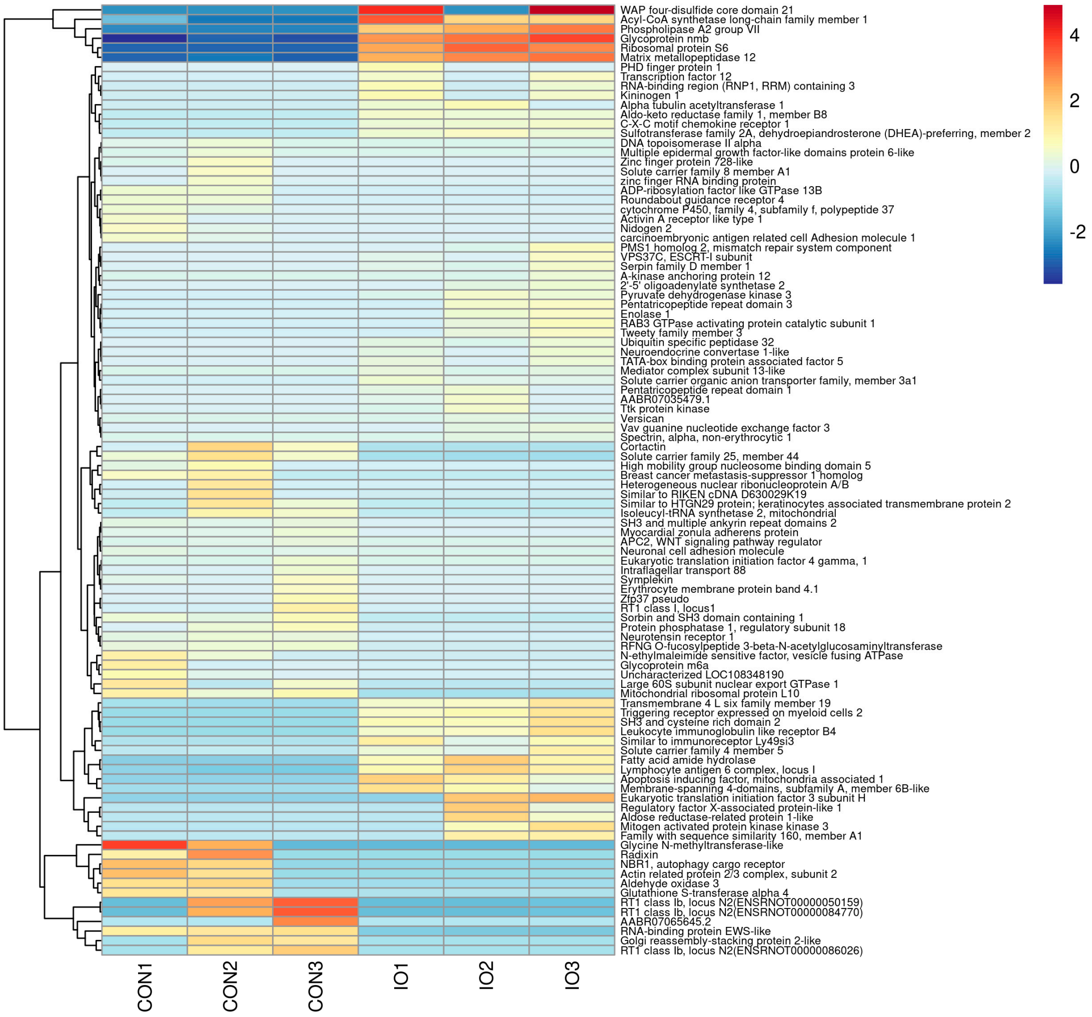

Figure S6. Hierarchical clustering heatmap of the top 50 up-regulated and top 50 down-regulated mRNAs. CON: control, IO: iron overload, n=3.

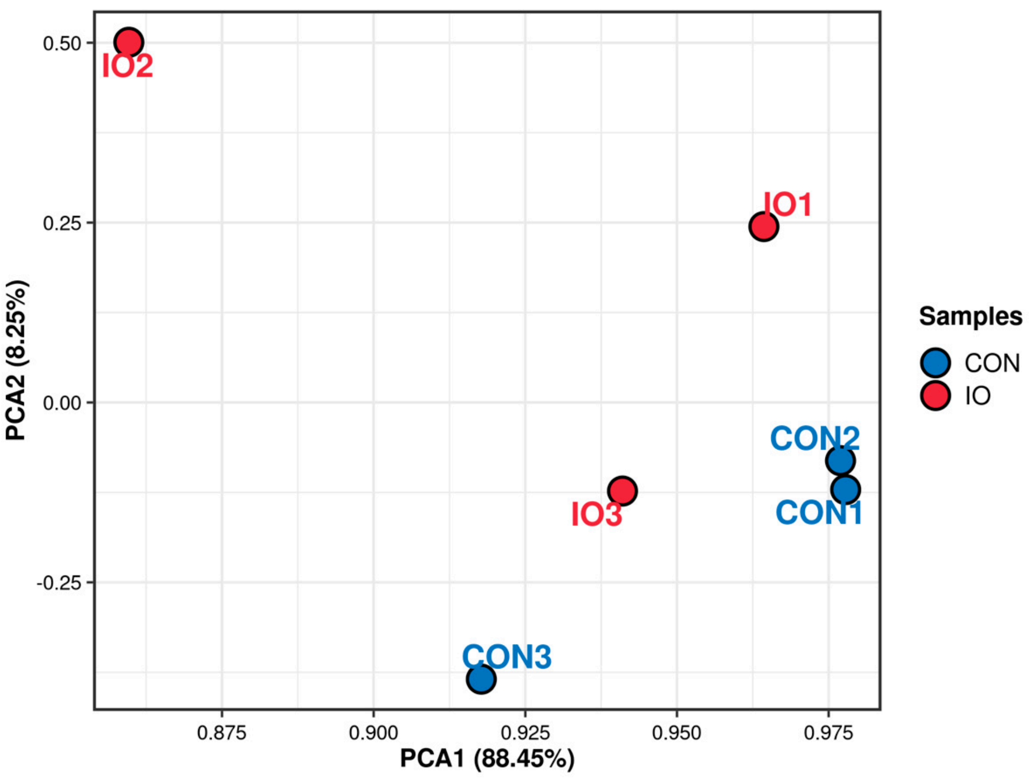

Figure S7. Principal components analysis (PCA) of LncRNAs. CON: control, IO: iron overload, n=3.

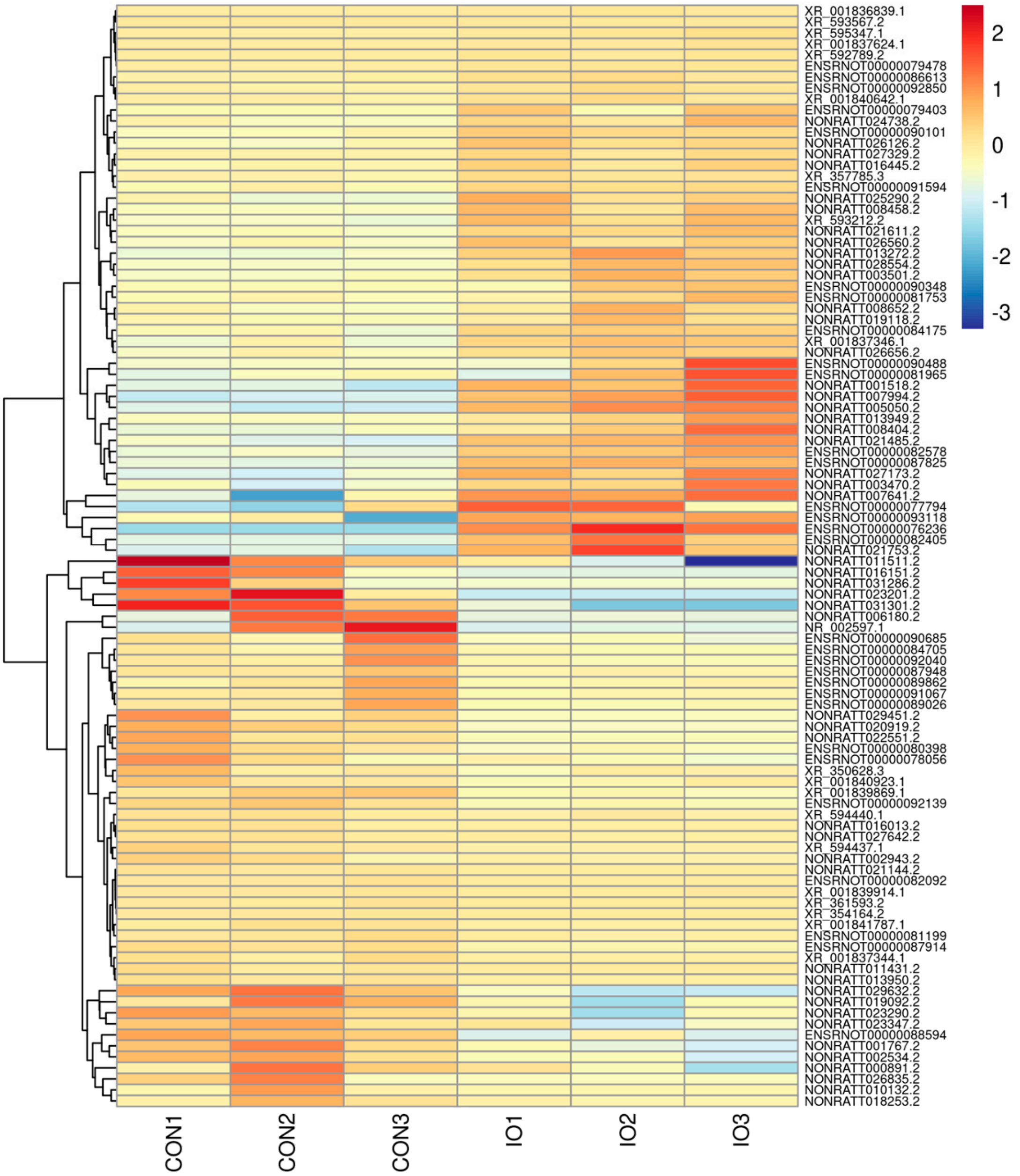

Figure S8. Hierarchical clustering heatmap of the top 50 up-regulated and top 50 down-regulated LncRNAs. CON: control, IO: iron overload, n=3.

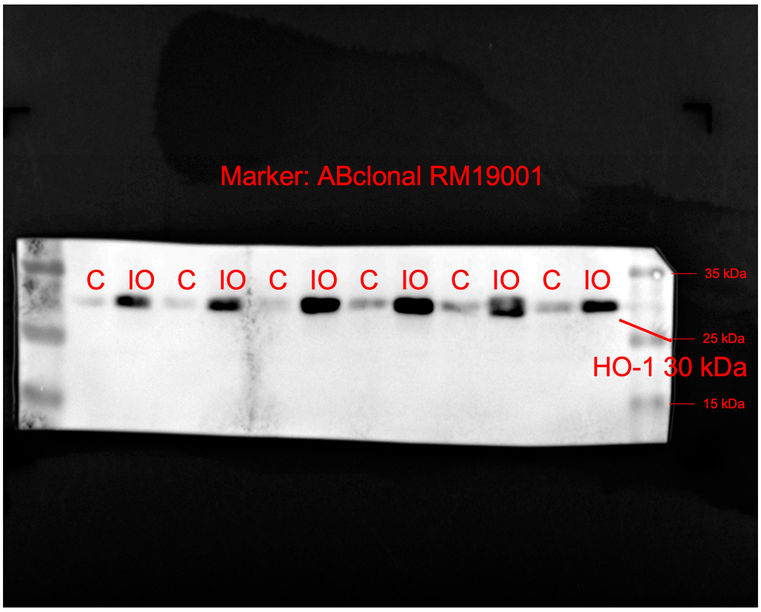

Figure S9. Original western blot figure of HO-1 in Figure 1L.

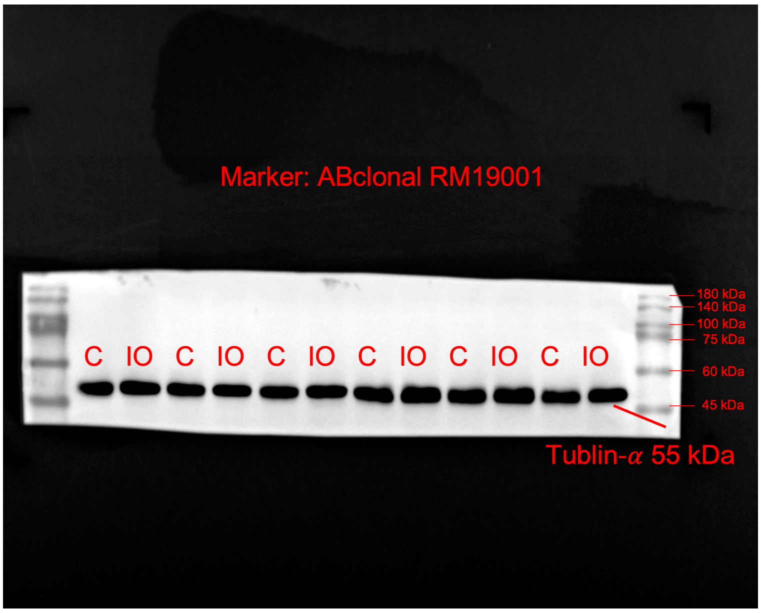

Figure S10. Original western blot figure of Tublin-α in Figure 1L.

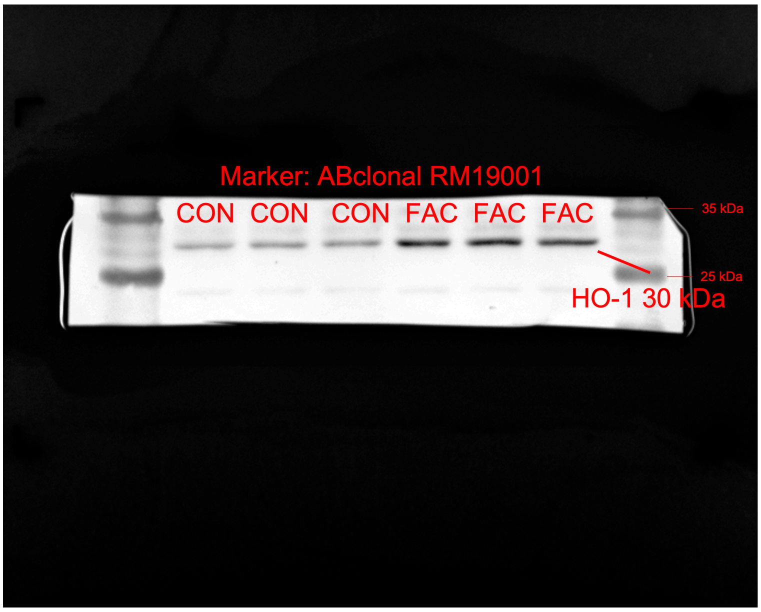

Figure S11. Original western blot figure of HO-1 in Figure 3K.

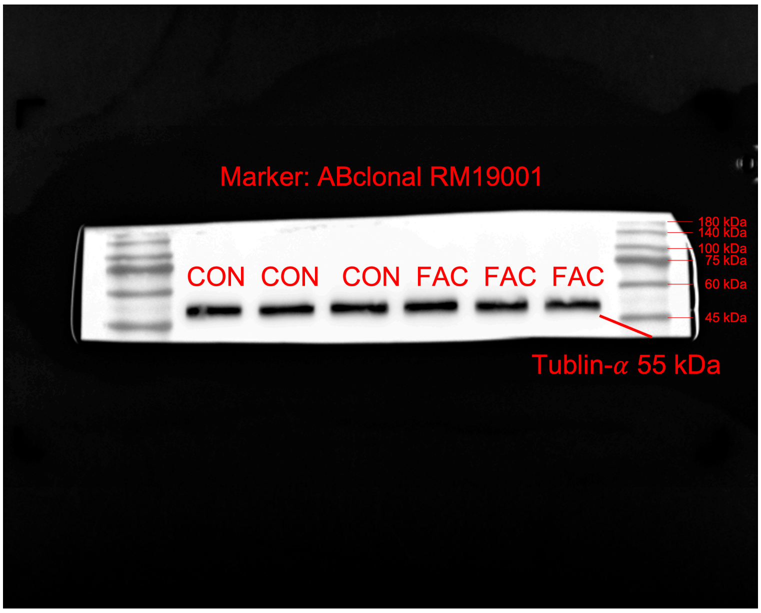

Figure S12. Original western blot figure of Tublin-α in Figure 3K.

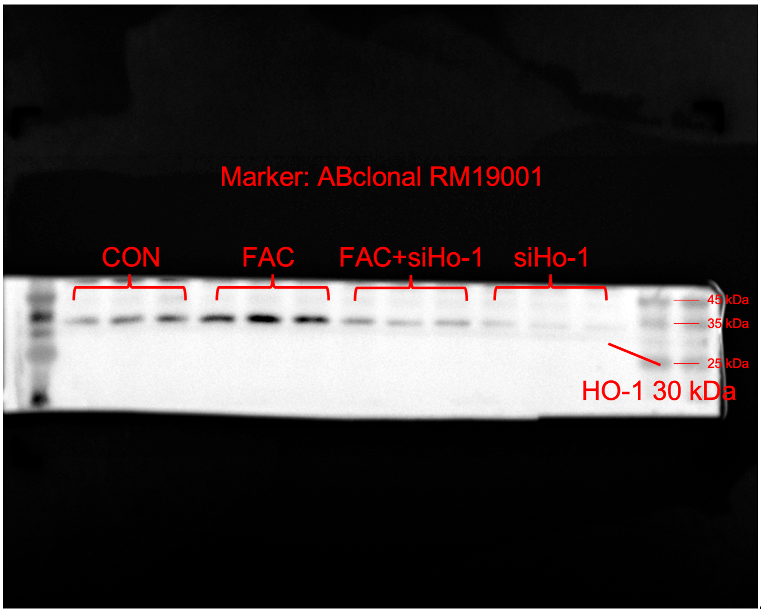

Figure S13. Original western blot figure of HO-1 in Figure 4B.

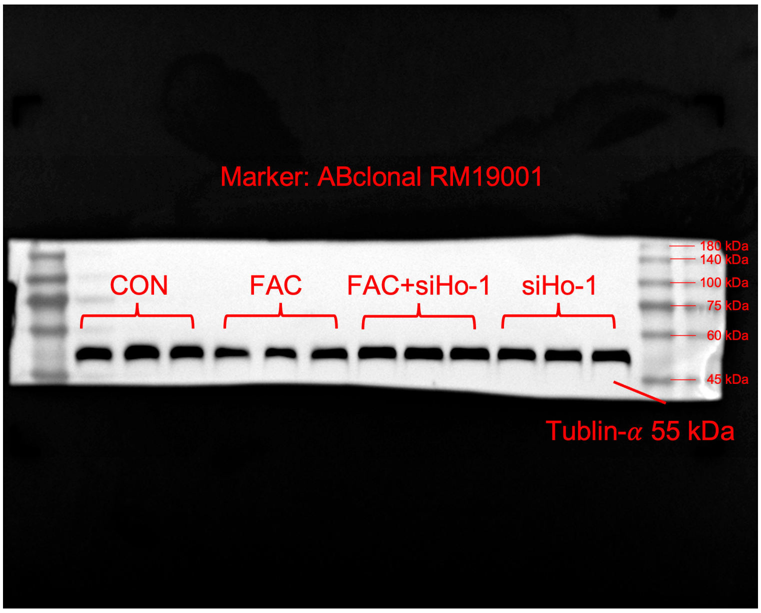

Figure S14. Original western blot figure of Tublin-α in Figure 4B.

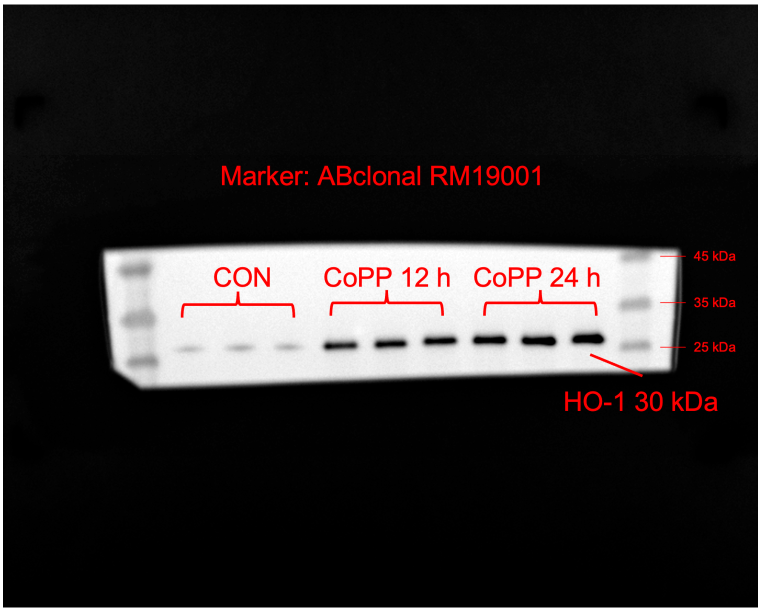

Figure S15. Original western blot figure of HO-1 in Figure 5E.

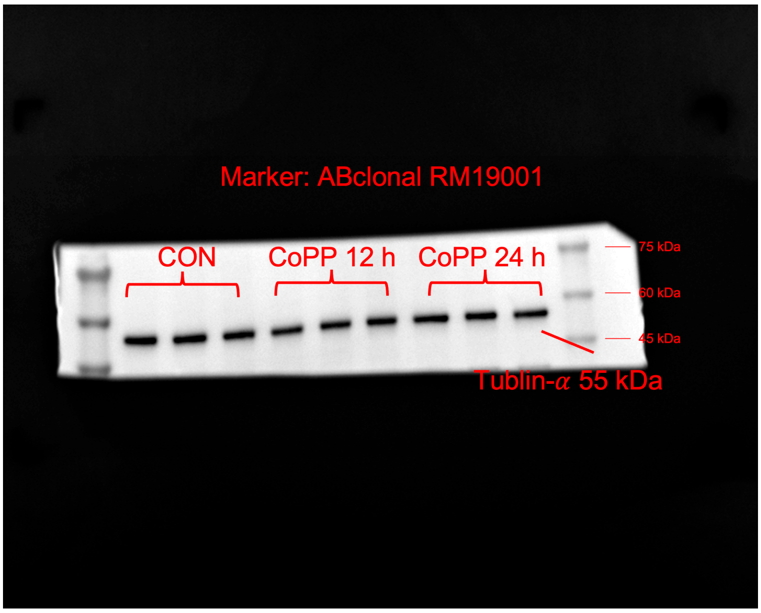

Figure S16. Original western blot figure of Tublin-α in Figure 5E.

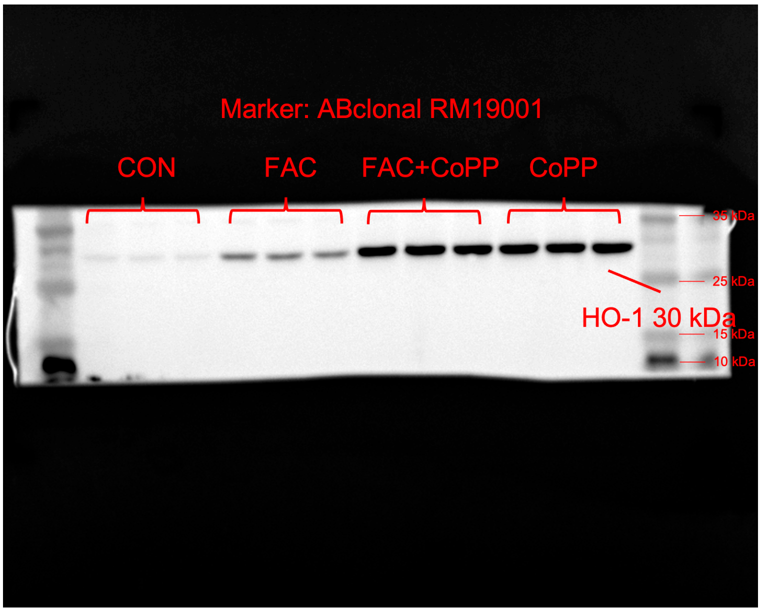

Figure S17. Original western blot figure of HO-1 in Figure 5I.

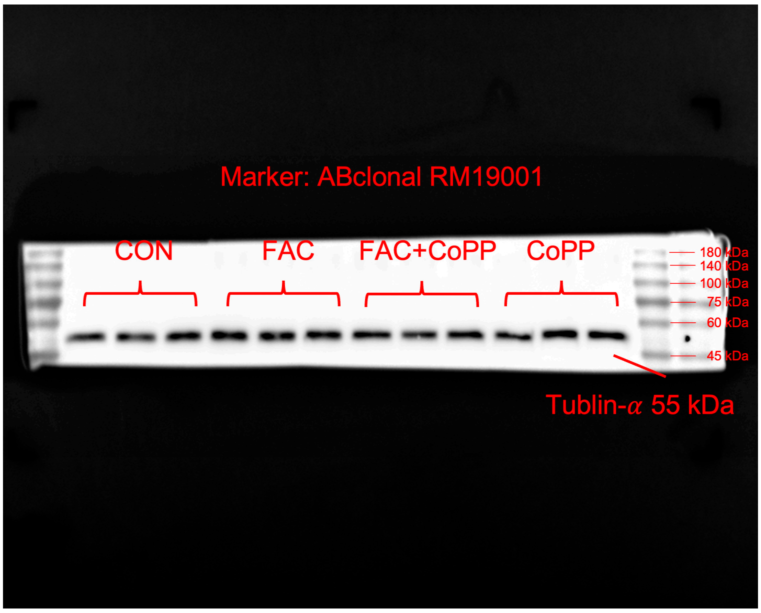

Figure S18. Original western blot figure of Tublin-α in Figure 5I.

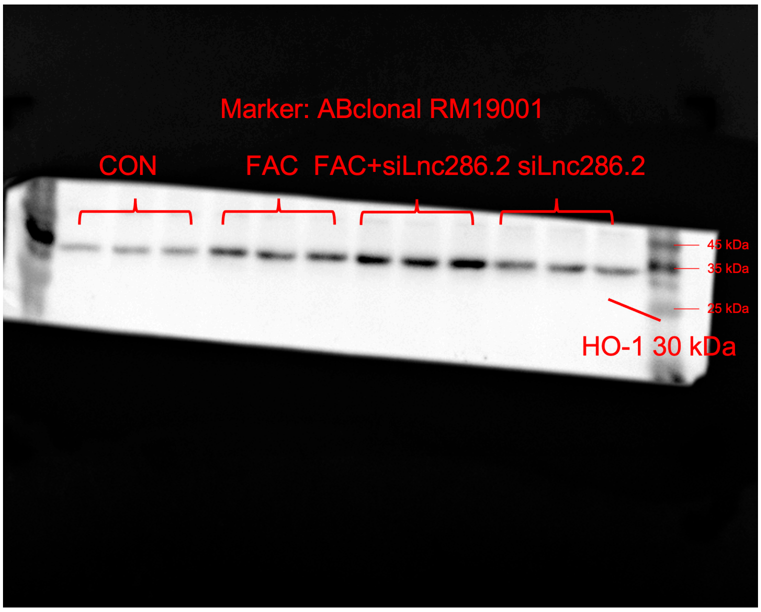

Figure S19. Original western blot figure of HO-1 in Figure 6C.

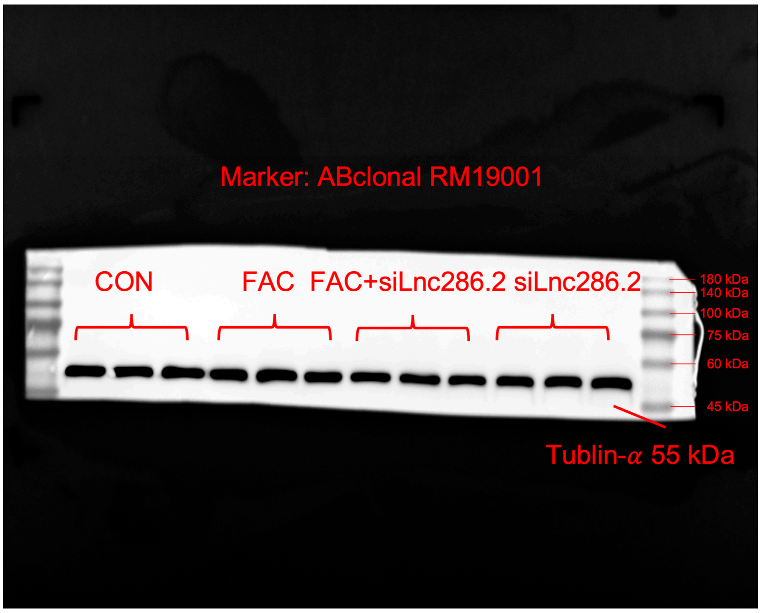

Figure S20. Original western blot figure of Tublin-α in Figure 6C.
